# Do people with hereditary cancer syndromes inform their at-risk relatives? A systematic review and meta-analysis

**DOI:** 10.1016/j.pecinn.2023.100138

**Published:** 2023-02-17

**Authors:** Muhammad Danyal Ahsan, Sarah R. Levi, Emily M. Webster, Hannah Bergeron, Jenny Lin, Priyanka Narayan, Becky Baltich Nelson, Xuan Li, Rana K. Fowlkes, Jesse T. Brewer, Charlene Thomas, Paul J. Christos, Eloise Chapman-Davis, Evelyn Cantillo, Kevin Holcomb, Ravi N. Sharaf, Melissa K. Frey

**Affiliations:** Weill Cornell Medicine, New York, NY, USA

**Keywords:** Disclosure, Cascade genetic testing, Hereditary cancer syndromes, Lynch syndrome, Hereditary breast and ovarian cancer

## Abstract

**Purpose:**

To evaluate rates of familial disclosure of hereditary cancer syndrome information.

**Methods:**

A systematic review and meta-analysis was conducted in accordance with PRISMA guidelines (PROSPERO no.: CRD42020134276). Key electronic databases were searched to identify studies evaluating hereditary cancer syndrome cascade relative disclosure. Eligible studies were subjected to meta-analysis.

**Results:**

Thirty-four studies met inclusion criteria. Among 11,711 included relatives, 70% (95% CI 60 - 78%) were informed of their risk of carrying a cancer-associated pathogenic variant; of 2,875 relatives informed of their risk who were evaluated for uptake of cascade testing, 43% (95% CI 27 - 61%) completed testing. Rates of disclosure were higher among female vs male relatives (79% [95% CI 73% - 84%] vs 67% [95% CI 57% - 75%]) and first-degree vs second-degree relatives (83% [95% CI 77% - 88%] vs 58% [95% CI 45 – 69%]).

**Conclusion:**

Nearly one-third of at-risk relatives remain uninformed of their risk of carrying a cancer-associated pathogenic variant. Even among those informed, fewer than half subsequently complete genetic testing, representing a critical missed opportunity for precision cancer prevention.

**Innovation:**

Five studies evaluating interventions to improve disclosure rates were generally ineffective. Urgent work is needed to elucidate barriers to relative disclosure by probands to develop targeted interventions that can optimize proband-mediated cascade genetic testing rates.

## Highlights

• 30% relatives are unaware of their risk of having a cancer associated pathogenic variant.
• Female relatives and first-degree relatives are more likely to be informed.
• Among those aware of their risk, only 43% complete genetic testing.

## 1 Introduction

Cascade genetic testing refers to the process of extending genetic testing to relatives of probands in whom germline pathogenic variants have been identified. In the context of cancer-predisposing pathogenic variants, cascade genetic testing offers the opportunity for cancer surveillance and risk-reduction strategies that can decrease cancer morbidity and mortality [[1], [2], [3], [4]]. There are several hereditary cancer syndromes with evidence-based surveillance guidelines to reduce cancer risk [5,6]. Furthermore, the Centers for Disease Control and Prevention Office of Public Health Genomics has designated cascade genetic testing for hereditary breast and ovarian cancer as well as Lynch syndrome as a tier one genomic application, defined as having significant potential for positive impact on public health [7]. Risk-reducing bilateral salpingo-oophorectomy and bilateral mastectomy are associated with a decreased risk of breast and ovarian cancer in individuals with *BRCA1/2* pathogenic variants, with risk-reducing bilateral salpingo-oophorectomy associated with a significantly lower all-cause mortality rate in this population [2]. For individuals with Lynch syndrome, surveillance with colonoscopy has been shown to decrease the risk for colorectal cancer, prevent colorectal cancer deaths, and decrease overall mortality [3,4].

Approximately four million people currently living in the United States harbor a cancer-associated pathogenic variant; however, the majority of these individuals are not aware [[8], [9], [10]]. Prediction modeling suggests that genetic testing at time of cancer diagnosis combined with cascade genetic testing of 70% of first- and second-degree relatives could result in identification of all carriers in less than a decade [10,11]. However, the literature suggests that only 35% of at-risk relatives currently complete cascade genetic testing for cancer syndromes [12]. Low rates of cascade genetic testing represent a critical missed opportunity in oncology care. Additionally, the literature suggests that racial and ethnic minorities and those of low socioeconomic status experience even greater underutilization of all aspects of genetic services including cascade testing [12,13].

Although relatives can learn of the presence of a cancer-associated pathogenic variant in their family without being informed by the proband, for example via direct contact by healthcare providers, disclosure by probands is the most common method of information dissemination within families [11]. Disclosure of genetic risk information by probands to their relatives is the critical first step in initiating the process of proband-mediated cascade genetic testing. As such, we aimed to systematically review the literature about disclosure patterns among families with cancer-associated pathogenic variants and conduct a meta-analysis on the pooled rates of disclosure and uptake of cascade testing among relatives informed of their risk, which has not previously been reported.

## 2 Methods

### 2.1 Overview

This systematic review was conducted in accordance with the Preferred Reporting Items for Systematic Reviews and Meta-Analyses (PRISMA) guidelines and was preregistered with PROSPERO (registration no.: CRD42020134276) [14]. A comprehensive literature search was devised with the assistance of a librarian and conducted on July 23, 2021, using the following bibliographic databases with no limit on year of publication: Ovid MEDLINE (In-Process and Other Non-Indexed Citations and Ovid MEDLINE 1946 to present), Ovid EMBASE (1974 to present), and Cochrane Library (Wiley). No article type, date, or language restrictions were included in the search. Search concepts included: cascade screening, genetic counseling, and cancer. The full Ovid MEDLINE search strategy is available in Supplementary Table 1.

### 2.2 Inclusion and exclusion criteria

**Fig. 1:**
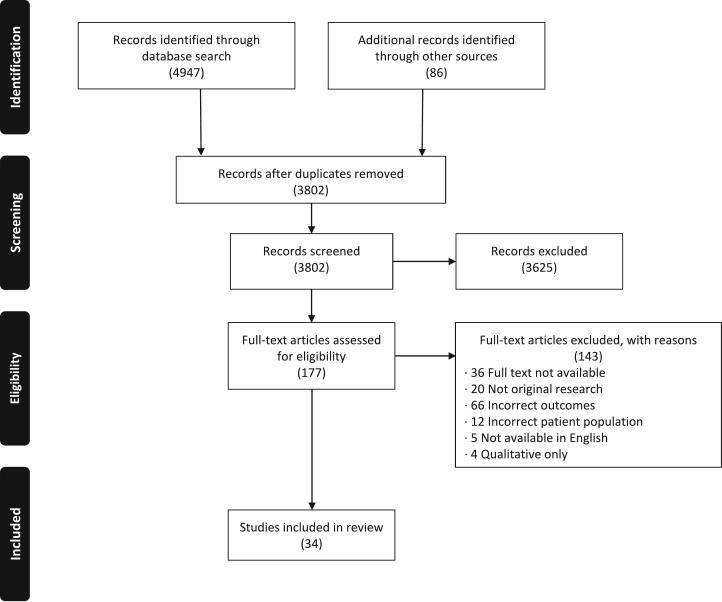
PRISMA Flow Diagram.

Eligible manuscripts included all primary English language studies that assessed cascade genetic testing for cancer-associated pathogenic gene variants with a focus on disclosure of genetic testing results to at-risk relatives. All non-primary research studies including commentaries, case reports, systematic reviews, and meta-analyses were excluded. A comprehensive review of reasons for exclusion of studies can be found in the PRISMA flow diagram (Figure 1).

### 2.3 Data extraction

All manuscripts were independently evaluated for inclusion by two reviewers and disagreements were discussed with a third reviewer. Data were independently extracted by two different reviewers, with a third reviewer checking the final extracted data for accuracy.

### 2.4 Risk of bias assessment

All included studies were evaluated for risk of bias in their design, conduct, and analysis using the Joanna Briggs Institute’s Critical Appraisal tools [15].

### 2.5 Statistical analysis

Meta-analyses for the proportion of probands that informed at least one at-risk relative, the proportion of at-risk relatives who were successfully informed and proportion of at-risk relatives who completed genetic testing among those informed were conducted using R software (Version 3.6.1[07/05/19], R Foundation for Statistical Computing, Vienna, Austria). Statistical heterogeneity was tested through the chi-square test (i.e., Cochrane Q test), and a P value < 0.2 was used to indicate the presence of heterogeneity. Statistical heterogeneity was also assessed by the inconsistency statistic (I^2^). A random effects analysis was used to calculate pooled proportions. The random effects analysis is more conservative and allows for more variability in the individual study proportion estimates when generating the pooled proportion. The pooled proportion was calculated using the Freeman-Tukey Double arcsine transformation, and the 95% CI was calculated using the Clopper-Pearson interval. The DerSimonian-Laird estimator was used to estimate the between-study variance. For the outcome proportions of interest, the results of each study were expressed as binary proportions with exact 95% CIs. For each meta-analysis, a funnel plot was constructed and reviewed, displaying the study proportion against study precision, estimated by the standard error, to assess for publication bias.

## 3 Results

### 3.1 Study characteristics

A total of 34 publications of original research were included in our systematic review, of which, data from 31 publications were included in our meta-analyses. Five studies evaluated interventions to improve disclosure rates among families. Although data from intervention arms of all five studies were excluded from our meta-analysis of non-intervention studies to avoid biasing results, two intervention studies utilized historical controls for comparison, data from which were included in our meta-analysis of non-intervention studies. Across all 34 included articles, study designs included 24 cross-sectional studies, 9 prospective studies, and 1 retrospective study. Study publication dates ranged from 2003-2020 and spanned 10 countries: United States (20), France (3), Australia (3), Netherlands (2), Belgium (1), Finland (1), Israel (1), Malaysia (1), Sweden (1), and the United Kingdom (1). Twenty studies evaluated disclosure rates among relatives at risk for hereditary breast and ovarian cancer only, 4 evaluated disclosure rates among those at risk for Lynch syndrome only, 1 among those at risk for hereditary pancreatic cancer only, and 9 included mixed hereditary cancer syndrome populations (Table 1).

**Table 1:** Demographics.*, **

Study	Hereditary Cancer Type (Specific Genes)	Method of Obtaining Disclosure Information	No. of Probands/ Relatives (degree of relatives)	Proband Age, years / Relative Age, years	Proband Sex, No. / Relative Sex, No.	Proband Cancer History, No. / Relative Cancer History, No.	Proband Race and Ethnicity, No. / Relative Race and Ethnicity, No.
Aktan-Collan et al, 2011[37]	Lynch Syndrome	Proband Self Report (Questionnaire)	248/0 (First – Children)	Probands: Mean: 56.4	Probands: Female: 127 Male: 121	Probands: Yes: 133	
Alegre et al, 2019[47]	HBOC**, Lynch Syndrome	Proband Self Report (Interview)	103/0	Probands: Mean: 55.2	Probands: Female: 92 Men: 11	Probands: Yes: 98 No: 5	
Bednar et al, 2020[16]	HBOC**, Lynch Syndrome, Other (*SDHB*, *SDHC, BAP1, PTEN, AXIN2, APC, NF1, TP53, VHL*)	Proband Self Report (Survey)	150/825 (First)	Probands: Mean: 46.2	Probands: Female: 132 Male: 18 Relatives: Female: 380 Male: 445		Probands: White: 140 Black/African American: 2 American Indian/Alaska Native: 2 Asian Indian: 1 Chinese: 1 Others: 4 Non-Hispanic: 139 Hispanic: 10 Prefer not to answer: 1
Blandy et al, 2003[17]	HBOC**	Proband Self Report (Interview)	30/310 (First, Second, Third)	Probands: Mean: 52.0	Probands: Female: 30 Relatives: Female: 162 Male: 148	Probands: Yes: 30 (breast and ovarian)	
Bradbury et al, 2007[28]	HBOC**	Proband Self Report (Interview)	42/86 (First – Children)	Probands: Median: 45.0 Relatives: Median: 12	Probands: Female: 37 Male: 5 Relatives: Female: 53 Male: 33	Probands: Yes: 23 No: 19	Probands: White: 39 Black: 1 Hispanic: 2
Bradbury et al, 2012[50]	HBOC**	Proband Self Report (Interview)	253/505 (First – Children)	Probands: Median: 47.7 Relatives: Median: 17	Probands: Female: 241 Male: 12 Relatives: Female: 253 Male: 252	Probands: Yes: 169 No: 84	Probands: White: 232 Black: 13 Others: 8
Brooks et al, 2004[18]	HBOC**	Not Reported	0/384 (First, Second, Distant)		Relatives: Female: 202 Male: 182		
Cheung et al, 2010[24]	HBOC**	Proband Self Report (Survey)	1,103/0		Probands: Female: 1,103	Probands: Yes: 776 No: 327	Probands: White: 948 Asian: 66 Latina: 61 African American: 28
Conley et al, 2020[27]	HBOC**	Proband Self Report (Questionnaire)	149/0	Probands: Mean: 44.9	Probands: Female: 149		Probands: Black: 149
Wagner Costalas et al, 2003[51]	HBOC**	Proband Self Report (Survey)	162/444 (First)	Probands: Median: 50 Relatives: Median: 50	Probands: Female: 162 Relatives: Female: 204 Male: 240		Probands: White: 147 Unknown: 15
Dilzell et al, 2014[25]	Lynch Syndrome	Proband Self Report (Questionnaire)	50/0 (First, Second)	Probands: Mean: 47.0	Probands: Female: 33 Male: 9		Probands: White: 41 Native American: 2 African American: 1 Asian: 1 Hispanic: 0 Others: 0 Unknown: 5 Relatives: White: 20 Native American: 1 Hispanic: 1 Others: 0 Unknown: 2
Eijzenga et al, 2018[38]	HBOC**, Hereditary Colorectal Cancer	Proband Self Report (Survey)	305/0	Probands: Intervention mean: 53.1 Control mean: 54.4	Probands: Female: 228 Male: 77	Probands: Yes: 216 No: 86	
Fehniger et al, 2013[42]	HBOC**	Proband Self Report (Interview)	73/606 (First, Second)	Mean: 47.4	Relatives: Female: 241 Male: 202		Probands: African American: 7 Asian/Pacific Islander: 14 Hispanic: 17 White: 32 Mixed: 3 Relatives: White: 135 African American: 53 Asian/Pacific Islander: 117 Hispanic: 123 Mixed: 15
Finlay et al, 2008[19]	HBOC**	Proband Self Report (Questionnaire)	115/655 (First, Second)		Probands: Female: 83 Male: 32		Probands: Ashkenazi Jewish: 28 Non-Ashkenazi/White: 79 Unknown/White: 7 Others: 1
Forrest et al, 2008[30]*	HBOC**, Lynch Syndrome, MEN Type 1, Peutz-Jegher syndrome	Proband Self Report (Interview)	19/131	Probands: Intervention mean: 39.2 Control mean: 38.1 Relatives: Intervention mean: 49.4 Control mean: 42.0	Probands: Female: 12 Male: 7 Relatives: Female: 66 Male: 65		
Gaff et al, 2005[31]	Lynch Syndrome		12/0				
Garcia et al, 2020[32]	HBOC**	Proband Self Report (Questionnaire)	40/0	Probands: Preintervention cohort median: 63.0 Postintervention cohort: median 49.0	Probands: Female: 40	Probands: Yes (breast and ovarian)	Probands: Preintervention: Non-Hispanic White: 18 Non-Hispanic Black: 2 Postintervention: Non-Hispanic White: 17 Non-Hispanic Black: 1 Hispanic: 1 Unknown: 1
Griffin et al, 2020[20]	HBOC**, Lynch Syndrome (*BRCA1, BRCA2, MLH1, MSH2, MSH6, PMS2, RAD51D*)	Proband Self Report (Survey)	64/1,955	Probands: Mean: 53.0	Probands: Female: 60 Male: 4		Probands: White: 62 African American: 2
Hall et al, 2018[36]	Non-BRCA 1/2, Non-Lynch Syndrome (*MYH* [Monoallelic, Biallelic], *CHEK2, ATM, PALB2, APC, TP53, CDH1, NBN, RAD51C, PTEN, RAD51D,* Other: *SDHA, SDHB, CDKN24, MRE11A, RAD50, FLCN, FH, MEN1, RET, CEBPA, EGFR, BMPR1A, BARD1, NF2*)	Proband Self Report (Survey)	57/0	Probands: Median: 52	Probands: Female: 47 Male: 10	Probands: Yes: 39 No: 18	Probands^c^: Non-Hispanic/White: 38 Hispanic: 11 Asian: 7 Ashkenazi Jewish: 3 Native American: 2 African American: 1 Others: 2
Hayat Roshanai et al, 2010[33]	HBOC**, Hereditary Colorectal Cancer	Proband Self Report (Interview)	147/81		Probands: Female: 133 Male: 14 Relatives: Female: 57 Male: 24	Probands: Yes: 54 No: 93	
Healey et al, 2017[34]	HBOC**	Proband Self Report (Interview)	165/0		Probands: Female: 138 Male: 27		
Kardashian et al, 2012[39]	HBOC**	Proband Self Report (Survey)	19/198 (First, Second, Cousins)		Probands: Female: 19		Probands: White: 14 Hispanic: 2 African American: 1 South Asian/Indian: 1 Asian/Pacific Islander: 1 Ashkenazi Jewish (as subset of above): 3
Kegelaers et al, 2014[29]	HBOC**	Proband Self Report (Questionnaire)	99/0	Probands: Mean: 49	Probands: Female: 74 Male: 25		Probands: White or Ashkenazi Jewish: 99
Landsbergen et al, 2005[35]	HBOC**	Proband Self Report (Questionnaire)	50/0	Probands: Mean at study: 49 Mean at testing: 44			
Lieberman 2018[21]	HBOC**	Proband Self Report (Questionnaire)	595/0 (First, Second)	Probands: Mean: 52.0			Probands: Ashkenazi Jewish: 595
McGivern et al, 2004[22]	HBOC**	Proband Self Report (Survey)	38/803 (First, Second, Third)	Probands: Mean: 48.1	Probands: Female: 38		Probands: White: 37 Native American: 1
Montgomery et al, 2013[40]	HBOC**	Proband Self Report (Survey)	345/1,046 (First)	Probands: Mean: 48.5	Probands: Female: 345		Probands: White: 328 Others: 17
Patenaude et al, 2006[47]	HBOC**	Proband Self Report (Questionnaire)	273/0		Probands: Female: 273		Probands: White: 273
Peters et al, 2019[49]	Pancreatic ductal adenocarcinoma (*APC, ATM, BRCA1, BRCA2, CDKN2A, CHEK2, MUTYH, NF1, PALB2, RAD50, TP53*)	Proband Self Report (Survey)	104/466	Probands: Median: 67	Probands: Female: 49 Male: 55	Probands: Yes: 104	Probands: White: 82 (Among 99 who completed MICRA)
Ricker et al, 2018[52]	HBOC**, Lynch Syndrome, Other Heritable Cancers (*APC, BMPR1A, CDH1, CDK4, CDKN2A, GREM1, MUTYH, NBN, POLE, POLD1 PTEN, SMAD4, STK11*, and *TP53*)	Proband Self Report (Questionnaire)	136/0	Probands: Mean: 52.4	Probands: Female: 105 Male: 31	Probands: Yes: 103 No: 33	Probands: Non-Hispanic/White: 63 Hispanic: 56 Asian: 14 Black: 2 Others: 1
Stoffel et al, 2008[26]	Lynch Syndrome	Proband Self Report (Questionnaire)	174/0	Probands: Mean: 46.7	Probands: Female: 122 Male: 52	Probands: Yes: 106 No: 68	Probands: White: 157 Non-White: 16 Unknown/missing: 1
Taber et al, 2015[23]	HBOC**, Lynch Syndrome	Proband Self Report (Survey)	77/0	Probands: Median: 54.5	Probands: Female: 58 Male: 17	Probands: Yes: 33 No: 44 (breast and colon)	Probands: Non-Hispanic White: 40 Non-Hispanic Black: 15 Hispanic/Latino: 13 Others: 3
Troian et al, 2020[48]	HBOC**	Proband Self Report (Questionnaire)	230/465 (First – Children)	Probands: Female mean: 48.8 Male mean: 60.4	Probands: Female: 160 Male: 70 Relatives: Female: 249 Male: 216	Probands: Yes: 44	
Yoon et al, 2011[53]	HBOC**	Not Reported	37/471 (First)	Probands: Median: 45.0	Probands: Female: 37 Relatives: Female: 227 Male: 244		Probands: Malaysian: 6 Indian: 8 Chinese: 23 Relatives: Malaysian: 11 Indian: 8 Chinese: 42

* Data for X-linked conditions and balanced reciprocal translocations from Forrest 2008 were excluded from our review.

** HBOC : Hereditary breast and ovarian cancer.

c Reported as per original article.

### 3.2 Cumulative patient characteristics

A total of 3,779 probands and 11,711 relatives were evaluated for disclosure of genetic test results. The median age of probands across all studies was 50 years and the median age of relatives across all studies was 33.5 years. Among 31 studies that included information on proband biologic sex, 5,118 (83.9%) were female and 981 (16.1%) were male. Among 11 studies that included information on relative biologic sex, 2,094 (50.5%) were female and 2,051 (49.5%) were male.

Twenty-two studies reported information on probands’ race and ethnicity. Among the 2,136 probands in these studies, 1601 (75.0%) were White, 224 (10.5%) were Black, 170 (8.0%) were Hispanic, 125 (5.9%) were Asian and 6 (0.3%) were Native American. Of these probands, 626 (29.3%) were Ashkenazi Jewish.

### 3.3 Cumulative rates of results disclosure and cascade genetic testing

**Fig. 2:**
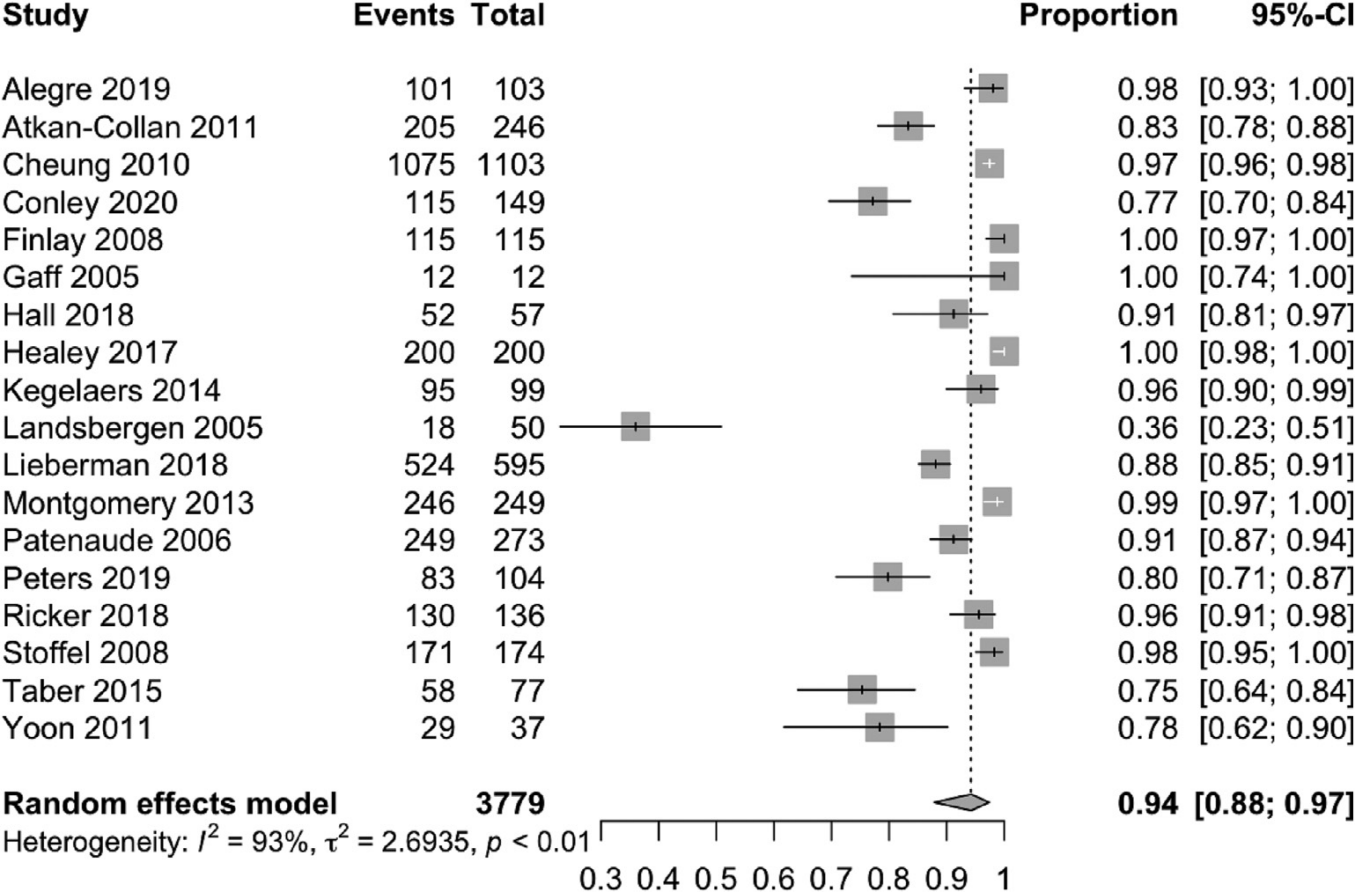
Pooled proportion of probands who informed at least 1 at-risk relative.

**Fig. 3:**
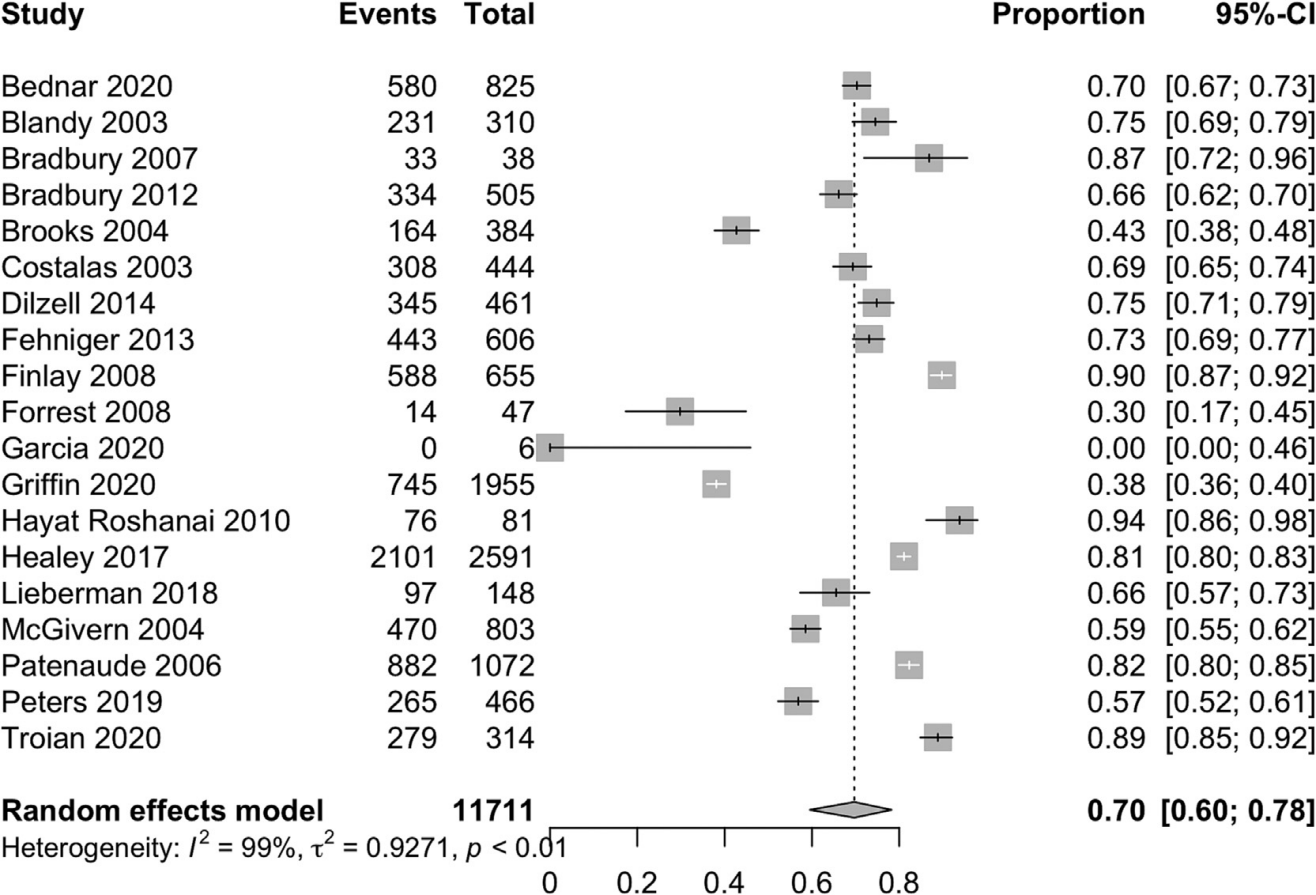
Pooled proportion of at-risk relatives who were informed.

**Fig. 4:**
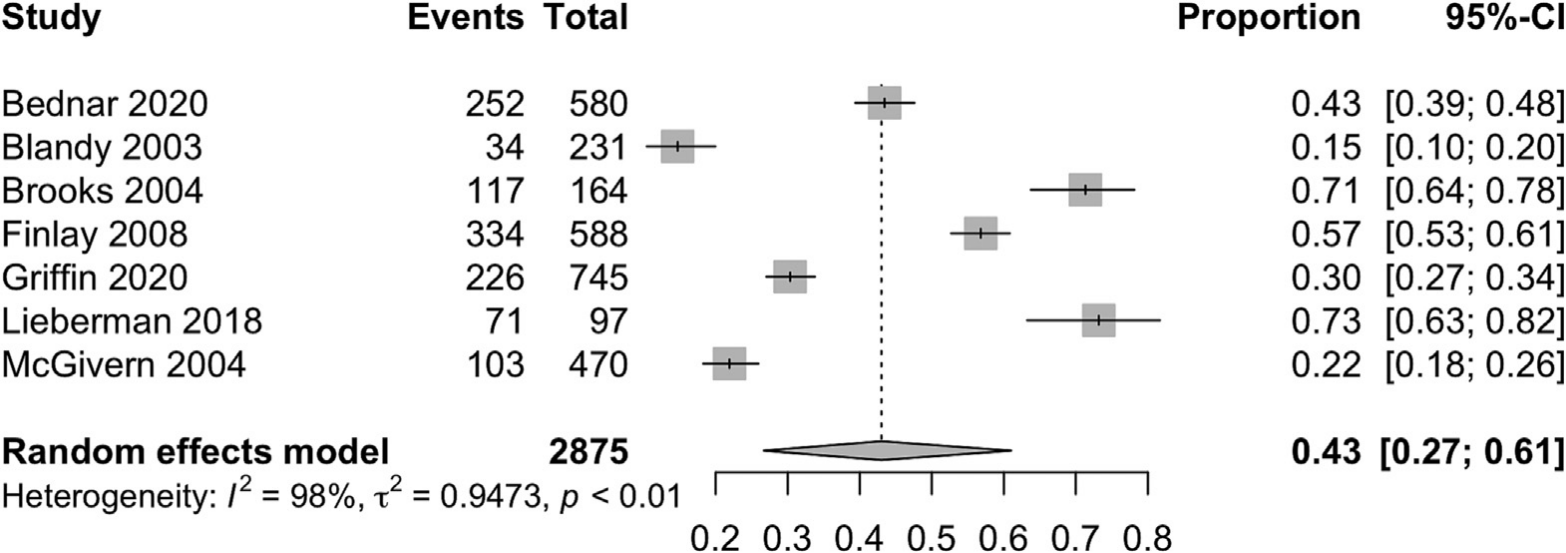
Pooled proportion of at-risk relatives who completed genetic testing among those informed.

Among 3,779 probands, 94% (95% CI 88-97%) initiated the cascade of information about their pathogenic gene variant by disclosing to at least one relative (Figure 2). Among 11,711 relatives, 70% (95% CI 60 - 78%) received information about the hereditary cancer syndrome identified in their family (Figure 3). Female relatives were more likely to have genetic information disclosed to them as compared to male relatives (79% [95% CI 73% - 84%] vs 67% [95% CI 57% - 75%]). First-degree relatives were also more likely to be informed as compared to second-degree relatives (83% [95% CI 77% - 88%] vs 58% [95% CI 45% – 69%]). Among 2,875 relatives across 7 studies who were informed of their risk of carrying a pathogenic gene variant, 43% (95% CI 27 - 61%) eventually completed genetic testing (figure 4) [[16], [17], [18], [19], [20], [21], [22]].

### 3.4 Additional factors associated with disclosure

Other studies identified factors associated with disclosure of genetic test results, but the data were either too limited or heterogenous to be meta-analyzed. Four studies reported on the association between race and ethnicity and results disclosure. Taber *et al.* and Cheung *et al.* reported higher rates of disclosure among White vs non-White families, whereas two studies found no association [[23], [24], [25], [26]]. Conley *et al.* studied disclosure patterns among Black families only, finding that 77% of Black probands in their cohort disclosed their genetic test results to at least one relative [27].

Five studies evaluated the impact of proband education level on rates of disclosure. Two studies reported that probands who had at least a college education were more likely to disclose results to relatives when compared to probands whose highest level of education was high school [28,29]. Three studies reported no association between proband education level and disclosure rates [21,25,26].

Two studies evaluated the impact of proband socioeconomic status on disclosure rates. Taber *et al.* reported that probands with annual incomes higher than $35,000 were more likely to share their genetic test results with relatives when compared to probands with annual incomes lower than $35,000; Cheung *et al.* reported no association between income and disclosure rates of probands [23,24].

### 3.5 Barriers to disclosure

Sixteen studies reported on barriers that probands faced in disclosing to relatives. The most common barrier to disclosure reported by probands in 10 studies was not being in close contact with relatives, including being estranged and not having relatives’ contact details [[20], [21], [22],^26^,[30], [31], [32], [33], [34], [35]]. Nine studies reported probands’ fear of causing their relatives distress or anxiety as a barrier to disclosure [[19], [20], [21], [22],[26], [27], [28],^30^,^35^]. Six studies reported that probands felt their relatives were either too old or too young to learn of the familial pathogenic variant [17,20,28,32,33,36]. Five studies reported that probands either did not know why it was important to share information about the familial pathogenic variant with relatives or that they felt genetic information was too personal to share [19,20,22,28,30]. Four studies reported probands did not disclose to relatives because they found the topic too distressing to bring up [19,27,30,37]. Other less commonly reported barriers included inferring a lack of interest from relatives and not feeling comfortable sharing complex medical information [19,21,22].

### 3.6 Interventions

Five studies evaluated interventions to assist probands in disclosing information about a familial pathogenic variant to their relatives (Table 2). A meta-analysis of disclosure rates among intervention studies was not possible due to an inadequate number of studies evaluating sufficiently homogenous interventions and reported outcomes. Two studies evaluated telephone counselling interventions whereby a member of the clinical team counseled the proband via telephone regarding identification of at-risk relatives, underscoring the importance of disclosing results, and providing information about what content to disclose [30,38]. Two studies evaluated the provision of written educational materials such as information about their familial cancer syndrome, information about cost and insurance coverage of genetic testing and letters to share with relatives informing them of their risk [32,39]. One study evaluated in-person counseling of probands to provide them with strategies on how to disclose to relatives, prepare them for relatives’ emotional reactions and share genetic counseling resources with their relatives [40]. Of all these studies, only Forrest et al and Kardashian et al reported positive results of their telephone counseling and written educational resource interventions respectively [30,39].

**Table 2:** Description of intervention studies.

Study	Design	Description of Intervention	Control Group (Non-Randomized Studies)	Disclosure rate in intervention group	Disclosure rate in control group	p-value
Eijzenga, 2018[38]	Randomized controlled trial	Telephone counselling of probands to assist them in disclosing	N/A	1^st^ Degree Relatives: 82% 2^nd^ Degree Relatives: 75%	1^st^ Degree Relatives: 83% 2^nd^ Degree Relatives: 78%	1^st^ Degree Relatives: NS 2^nd^ Degree Relatives: NS
Forrest, 2008[30]	Non-randomized pre-post study	Telephone counselling of probands to assist them in disclosing	Historical controls	61%	36%	p=0.01
Garcia, 2020[32]	Non-randomized pre-post study	Provision of written educational materials to probands	Historical controls	77%	83%	p=0.26
Kardashian, 2012[39]	Non-randomized pre-post study	Provision of written educational materials to probands	All eligible patients seen for genetic counselling prior to implementation of intervention	1^st^ Degree Relatives: 90% 2^nd^ Degree Relatives: 75% Cousins: 63%	1^st^ Degree Relatives: 88% 2^nd^ Degree Relatives: 38% Cousins: 40%	1^st^ Degree Relatives: p=1.00 2^nd^ Degree Relatives: p=0.32 Cousins: p=0.86
Montgomery, 2013[40]	Randomized controlled trial	In-person counselling of probands to help them in disclosing	N/A	Shared with at least one relative: 99.3% Shared with all FDR: 54.0%	Shared with at least one relative: 99.2% Shared with all FDR: 52.7%	Shared with at least one relative: p=0.59 Shared with all FDR: p=0.83

### 3.7 Risk of bias assessment

**Supplementary Fig. 1:**
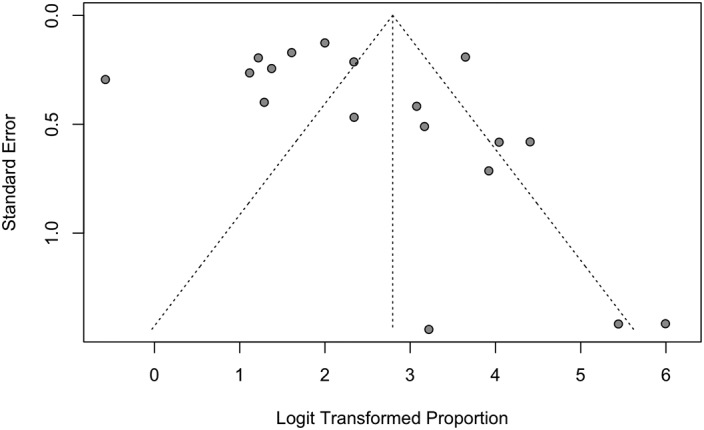
Funnel plot for pooled proportion of probands who Informed at least 1 at-risk relative

**Supplementary Fig. 2:**
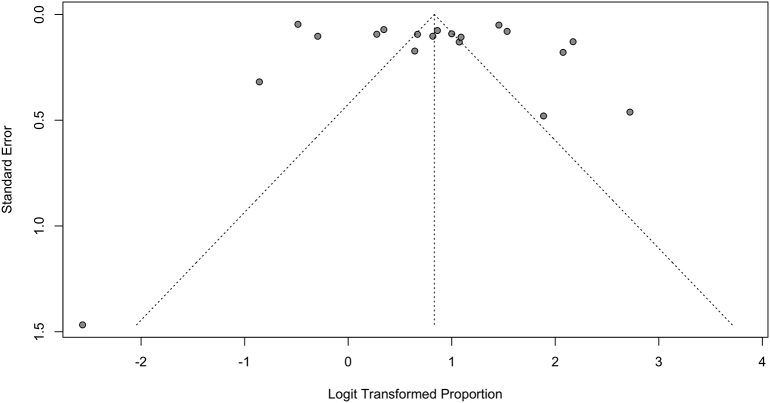
Funnel plot for pooled proportion of at-risk relatives who were informed

**Supplementary Fig. 3:**
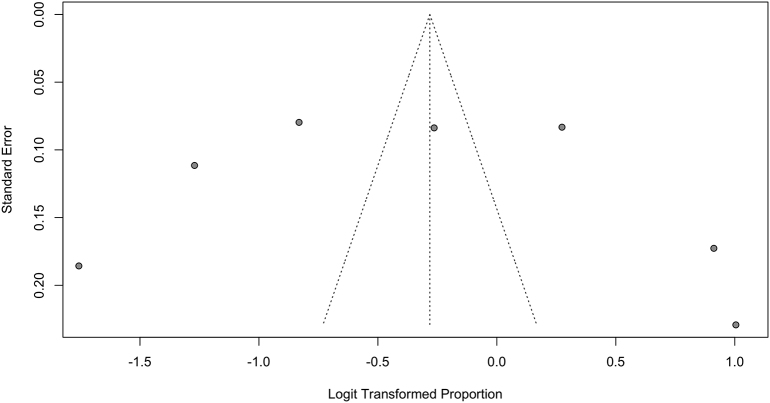
Funnel plot for pooled proportion of at-risk relatives who completed genetic testing among those informed

Studies were assessed for risk of bias using tools from the Joanna Briggs Institute. Although all studies suffered from risk of bias in at least one domain, most commonly in lack of identification and control of confounders, they were all deemed appropriate to include in this synthesis. The funnel plots for rates of disclosure and uptake of cascade genetic testing suggest underrepresentation of smaller studies (Supplementary Fig. 1, Supplementary Fig. 2, Supplementary Fig. 3).

## 4 Discussion

We have systematically reviewed the available literature on disclosure of genetic test results by probands to their relatives in the context of cancer-associated pathogenic variants and found that up to 30% of relatives are not aware of the familial cancer risk. Disclosure of genetic testing results by probands is the obligate prerequisite to the process of proband-mediated cascade genetic testing that can result in early cancer detection and cancer prevention for at-risk relatives. With the majority of cancer-associated pathogenic gene variant carriers in the U.S. unaware of their risk, determining rates of disclosure as well as uptake of genetic testing among those relatives to whom disclosure was made, are essential to characterize the efficiency of cascade genetic testing when the process is mediated by probands [9]. Subsequently, this will enable the field to identify avenues for improvement. To the best of our knowledge, this is the first meta-analysis on rates of disclosure among probands identified to have cancer-associated pathogenic gene variants to their at-risk relatives. Although our prior systematic review and meta-analysis reported uptake rates of cascade genetic testing, uptake rates specifically among relatives who were informed of their risk of carrying a pathogenic gene variant were not reported, and to the best of our knowledge, this is also the first meta-analysis reporting this outcome [12].

Notably, our findings revealed that only 70% of at-risk relatives are aware of their increased risk of carrying a cancer-associated pathogenic gene variant. Furthermore, among those relatives who are successfully informed, only 43% successfully complete genetic testing to define their pathogenic variant status. Probands were more likely to disclose to female vs male relatives, a trend that has also been observed in uptake of cascade genetic testing [12]. Probands were more likely to disclose to first-degree vs more distant relatives, which has also been reported for cascade testing [12]. Limited literature suggested that probands who were non-White, with lower income and lower levels of completed education were less likely to disclose genetic risk information to their relatives; however, other studies found no association. These studies evaluated the impact of race, ethnicity, income and education level on disclosure rates as secondary outcomes and could thus be underpowered for these outcomes. This highlights the need for well-designed studies, specifically evaluating the influence of these factors on relative disclosure rates. Literature on the impact of race, ethnicity, income and education on cascade testing is also sparse; however, limited data suggest similar trends of lower uptake rates among racial and ethnic minorities, relatives with lower income, and those with lower levels of completed formal education [24,[41], [42], [43], [44]]. Notably, 75% of probands among all studies that reported on disclosure of genetic test results were identified as White. Lack of inclusion of racially and ethnically diverse populations is a critical issue in cancer genetics research and highlights the need to study disclosure patterns among minority and underserved populations [41,45].

This study should be viewed in light of important limitations. First, disclosure of genetic test results to relatives was reported by probands and not confirmed by relatives for all included studies, and thus the meta-analyzed data are subject to recall bias. Furthermore, funnel plots for both proportion of probands disclosing to at least one at-risk relative as well as proportion of at-risk relatives disclosed to demonstrate a skew towards higher disclosure rates among smaller studies. However, larger studies far outnumbered smaller studies in both these funnel plots, and thus the absence of smaller studies with lower disclosure rates is unlikely to be a significant contributor to publication bias. Finally, although all studies were deemed to be of sufficiently low risk of bias overall to be included in our synthesis, no study was entirely unbiased—every included study suffered from risk of bias in one or more domain, most commonly failing to identify and control for confounders.

### 4.1 Innovation

Interventions aimed to better equip probands to disclose genetic risk information by providing written or telephone resources were generally ineffective, highlighting the need for studies focused on proband barriers to disclosure so that targeted interventions can be developed.

Recent studies have focused on direct relative contact via clinicians or genetic testing clinical laboratories as a strategy to increase rates of cascade genetic testing [11,12]. Direct relative contact is promising in alleviating barriers such as strained relationships, lack of contact and distress in disclosing to relatives, which we found to be the most commonly reported barriers to disclosure. However, our review suggests that several other factors may contribute to an individual’s decision to disclose results to their at-risk relatives including biologic sex, degree of relation and family demographics. It is thus of vital importance to further characterize patterns in and barriers to disclosure to relatives of cancer genetic information in order to develop innovative interventions that can result in equitable improvement in familial disclosure and completion of cascade cancer genetic testing.

The following are the supplementary data related to this article.

Supplementary Table 1 Search Strategy Supplementary Table 1

## Funding support

Melissa K. Frey is supported by the following grant: NIH/NCATS Grant # KL2-TR-002385. Ravi N. Sharaf is supported by the following grants: National Cancer Institute Grant # K07CA216326 and R01CA211723 and Patient Centered Outcomes Research Institute Grant # IHS-2017C3-9211.

Paul J. Christos and Charlene Thomas are supported by the following grant: 10.13039/100007273Clinical and Translational Science Center at Weill Cornell Medical College (1-UL1-TR002384-01).

## Declaration of Competing Interest

Kevin Holcomb reports a relationship with Johnson & Johnson that includes: consulting or advisory.
