# Supplementary material for: Do people with hereditary cancer syndromes inform their at-risk relatives? A systematic review and meta-analysis
Source: PEC Innov. 2023 Feb 17;2:100138. doi: 10.1016/j.pecinn.2023.100138 (PMC10194207; doi:10.1016/j.pecinn.2023.100138)
Supplement: Supplementary Table 1 — Search Strategy [file mmc4.docx]

| **Ovid Medline:** |
| --- |
| 1. genetic counseling/ or ((genetic adj3 counseling) or (genetic adj3 counselling) or (preventative adj3 genetic*)).ti. |
| 2. genetic disorder/ or ((genetic adj3 defect*) or (genetic adj3 disease*) or (genetic adj3 disorder*) or (genetic adj3 syndrome*) or (hereditary adj3 defect*) or (hereditary adj3 disease*) or (hereditary adj3 disorder*) or (hereditary adj3 syndrome*) or (heredodegenerative adj3 defect*) or (heredodegenerative adj3 disease*) or (heredodegenerative adj3 disorder*) or (heredodegenerative adj3 syndrome*) or (single-gene adj3 defect*)).ti. |
| 3. exp genetic predisposition/ or ((genetic adj3 anticipation) or (genetic adj3 predisposition*) or (genetic adj3 prognos*) or (genetic adj3 resistance) or (genetic adj3 susceptibilit*)).ti. |
| 4. genetic screening/ or genetic carrier screening/ or ((genetic adj3 test*) or (genetic adj3 screen*)).ti. |
| 5. or/1-4 |
| 6. 5 and cascad*.ti. |
| 7. ((cascade adj5 test*) or (cascade adj5 screen*) or (famil* adj5 test*) or (famil* adj5 screen*) or (hereditary adj5 test) or (hereditary adj5 screen*)).ti. |
| 8. 6 or 7 |
| 9. exp Neoplasm/ or (cancer* or carcino* or cyst* or leukemi* or lymphom* or malignan* or melanoma* or myeloma* or neoplas* or oncolog* or sarcoma* or tumor* or tumour*).ti. |
| 10. 8 and 9 |

Supplementary Table 1. Search strategy.
